# Reemergence of *Reston* *ebolavirus* in Cynomolgus Monkeys, the Philippines, 2015

**DOI:** 10.3201/eid2407.171234

**Published:** 2018-07

**Authors:** Catalino Demetria, Ina Smith, Titus Tan, Daniel Villarico, Edson Michael Simon, Rex Centeno, Mary Tachedjian, Satoshi Taniguchi, Masayuki Shimojima, Noel Lee J. Miranda, Mary Elizabeth Miranda, Melissa Marie R. Rondina, Rowena Capistrano, Amado Tandoc, Glenn Marsh, Debbie Eagles, Ramses Cruz, Shuetsu Fukushi

**Affiliations:** Research Institute for Tropical Medicine, Muntinlupa City, Philippines (C. Demetria, T. Tan, D. Villarico, E.M. Simon, R. Centeno, R. Capistrano, A. Tandoc III);; CSIRO Australian Animal Health Laboratory, East Geelong, Victoria, Australia (I. Smith, M. Tachedjian, G. Marsh, D. Eagles);; National Institute of Infectious Diseases, Tokyo, Japan (S. Taniguchi, M. Shimojima, S. Fukushi);; INA Research Philippines, Muntinlupa City (N.L.J. Miranda, M.E. Miranda, M.M.R. Rondina, R. Cruz)

**Keywords:** Reston Ebolavirus, viruses, nonhuman primates, measles virus, coinfection, macaques, zoonoses, the Philippines

## Abstract

In August 2015, a nonhuman primate facility south of Manila, the Philippines, noted unusual deaths of 6 cynomolgus monkeys (*Macaca fascicularis*), characterized by generalized rashes, inappetence, or sudden death. We identified *Reston* *ebolavirus* (RESTV) infection in monkeys by using serologic and molecular assays. We isolated viruses in tissues from infected monkeys and determined viral genome sequences. RESTV found in the 2015 outbreak is genetically closer to 1 of the 4 RESTVs that caused the 2008 outbreak among swine. Eight macaques, including 2 also infected with RESTV, tested positive for measles. Concurrently, the measles virus was circulating throughout the Philippines, indicating that the infection of the macaques may be a reverse zoonosis. Improved biosecurity measures will minimize the public health risk, as well as limit the introduction of disease and vectors.

*Reston* *ebolavirus* (RESTV) was discovered after an outbreak of hemorrhagic disease in cynomolgus macaques in a primate research facility in Reston, Virginia, USA in 1989 that had imported macaques from the Philippines (*1*). Subclinical infections in humans in the facility were determined through diagnostic testing. Other outbreaks of RESTV epizootics were identified in Sienna, Italy in 1992 (*2*); Alice, Texas, USA in 1993 (*3*); and 2 outbreaks in the Philippines in 1996; all 4 outbreaks involved purpose-bred cynomolgus macaques (*Macaca fascicularis*) attributed to a single nonhuman primate (NHP) facility in the Philippines (*4*). Until the 2015 outbreak described here, no outbreaks of RESTV had occurred in the Philippines since 1997; subsequently, the government permanently closed the facility.

The last known occurrence of RESTV epizootic in the Philippines was during 2008–2009 and affected 2 piggeries on the island of Luzon, 1 of the 3 major islands in the country. The disease was discovered as a co-infection with porcine reproductive and respiratory syndrome virus (PRRSV), also prevalent at that time (*5*). After this outbreak, Jayme et al. undertook a search for a possible reservoir in bats by using low levels of viral RNA detected in the microbat *Miniopterus schreibersii* (*6*).

As part of the established process for testing of macaques in the quarantine facility, animals that are sick or die are routinely tested for the presence of RESTV infection. In August 2015, six monkeys that were in the last stage of quarantine died suddenly; their bodies were submitted for testing. Although there are many fruit-bearing trees in the facility, the building was constructed in such a way that fruit bats could not make contact with the monkeys. However, rats were observed entering the cages of individual primates in the facility.

At the same time, an outbreak of measles virus (MV) was occurring among humans nationwide. During the first 6 months of 2015, there were 2,231 reported cases, of which 534 were laboratory-confirmed (*7*).

In this study, we describe the serologic and molecular detection of RESTV and MV from macaques in the quarantine facility in the Philippines in 2015, and demonstrate genetic characterization of the isolated RESTV.

## Materials and Methods

### Facility

The size of the monkey quarantine facility, located in the province of Southern Luzon, is ≈3,000 km^2^. The 174 monkeys sampled were housed in 2 separate buildings that are equipped with individual stainless steel squeeze cages measuring ≈58 × 48 × 77 cm^3^, arranged in 4 rows: the cages in the first and second rows, and those in the third and fourth rows face each other. Each building has its own anteroom and is surrounded by large windows that have screens and welded wire to protect the monkeys from vermin and prevent monkey escapes. Each cage is equipped with a lock and squeeze-back mechanism. There were separate personnel assigned to each building and each were required to wear individual personal protective equipment (undergarments, coveralls, mask, caps, goggles, socks, and boots) and to shower when entering and exiting the animal buildings. Materials, such as those used during animal care procedures, as well as cleaning implements, were not shared between buildings. We used new sterile disposable syringes with needles for each monkey and for each procedure. We disposed of used syringes and needles in dedicated containers in each building and disposed of them through a government-accredited waste contractor.

### Samples

Both antigen and antibody detection methods were used in the laboratory investigation of the epizootic occurrence. We collected a total of 174 samples from the facility for RESTV IgG and MV IgM screening. Blood samples were centrifuged on site and serum samples were transferred to labeled cryovials and transported through a cold chain. The serum samples were heat-inactivated at 56°C for 1 h. Spleen, liver, and lymph nodes from 4 deceased monkeys were also collected and transported in the same manner as the sera and tested for RESTV by using molecular assays.

Serum samples from macaques in the 2 breeding facilities located in Oriental Mindoro and Rizal that supplied the macaques to the quarantine facility were also tested for RESTV antibodies, as were 71 personnel in the facilities.

### RESTV Serologic Analysis

Indirect ELISA testing was used following the protocols of either the Centers for Disease Control and Prevention (CDC) (*8*) or the protocol of the National Institute of Infectious Diseases (Musashimurayama, Tokyo, Japan) reagents (*9*). Briefly, for the CDC protocol, the upper half of the ELISA plate (Falcon, Avenel, NJ, USA) was coated with gamma-irradiated antigens obtained from a RESTV-infected cell suspension, and the lower half with those obtained from a non-infected cell suspension. We added test samples to the wells, diluted 4-fold starting at 1:100. We used mouse anti-human IgG with horseradish peroxidase (Accurate Chemical and Scientific Company, Westbury, NY, USA), diluted at 1:4,000, as a secondary antibody. We added 2,2'-azino-bis (3-ethylbenzothiazoline-6-sulphonic acid substrate (Kirkegaard-Perry Laboratories, Gaithersburg, MD, USA) at the last step for visualization of the antigen-antibody reaction. The optical density value was recorded at 415 nm by using an ELISA plate reader (ThermoFisher Scientific, Carlsbad, CA, USA). Samples were considered reactive if the adjusted optical density (OD) was ≥0.95. For the National Institute of Infectious Diseases protocol, the upper half of the ELISA plate was coated with RESTV recombinant nucleoprotein (NP) tagged with glutathione S transferase, expressed in *Escherichia coli* at ≈100 ng/well, and the lower half with negative control glutathione S transferase antigen. Goat anti-human IgG conjugated with Novex horseradish peroxidase (ThermoFisher) diluted at 2 μg/mL was used as a secondary antibody. Samples were considered reactive if the sample showed an OD ≥0.56 at 1:100 dilution, or 0.23 at 1:400 dilution.

We retested all serologically reactive samples by using immunofluorescent assay (IFA) as described by Ikegami et al. (*10*). In brief, serum samples were 2-fold serially diluted in phosphate-buffered saline (PBS) from 1:10 to 1:640. Diluted serum (20 µL) was loaded onto each well of the IFA slide (14 wells; AR Brown Co., Ltd., Toyo, Japan) containing HeLa cells expressing RESTV recombinant NP. The slides were incubated for 1 h at 37°C and washed 3 times with PBS. Invitrogen Fluorescein isothiocyanate-labeled antibody against human IgG (ThermoFisher) diluted in PBS at 1:200 was added to each well and incubated for 1 h at 37°C. After washing with PBS, the slides were examined for the staining pattern under an immunofluorescent microscope (Nikon, Chiyoda, Japan) and their reactions were recorded.

In addition, we tested all reactive serum samples for antibodies against RESTV glycoprotein in a Luminex assay (Luminex Corporation, Austin, TX, USA). Briefly, Luminex beads coated with RESTV glycoprotein (Bead #35) were blocked in 2% skim milk Tween and phosphate-buffered saline (TPBS) for 30 min at room temperature in the dark with shaking in a flat-bottom microtiter plate. We washed the plate twice with TPBS by using a magnetic plate washer (BioPlex Pro II Wash Station; Bio-Rad, Hercules, CA, USA). Serum diluted (1:100, 100 µL) in TPBS was added and incubated for 30 minutes, as stated before. The plate was washed and 100 µL of a mixture of biotinylated Protein A (1:500)/biotinylated Protein G (1:250) (ThermoFisher Scientific, Brisbane, Queensland, Australia) was added to each well and incubated as described above. The plate was washed again, then 100 µL of streptavidin–phycoerythrin was added (1:1,000; Thermo Scientific, Brisbane, Queensland, Australia), and the plate was incubated as before. Samples were assayed on the BioPlex machine (Bio-Rad) and the median fluorescence intensity read for 100 beads.

### Measles Serologic Analysis

The 174 serum samples from macaques in the primate quarantine facility were tested for MV antibody by Enzygnost Measles Anti-IgM ELISA (Siemens Healthcare Diagnostics, Marburg, Hesse, Germany). We diluted the serum samples in rheumatoid factor adsorbent to remove inhibitors that might interfere with the reaction. We used the ELISA by following the manufacturer’s instructions. A sample was determined to be negative if the corrected OD at a wavelength of 450 nm with a reference at a wavelength of 635 nm was ≤0.100, equivocal if it was 0.101–0.199, and positive for MV IgM if it was ≥0.200.

### Molecular Detection of RESTV

We isolated total RNA from lymph node, liver, and spleen samples of infected animals by using a combined method of TRIzol inactivation and QIAGEN RNeasy Mini Kit (QIAGEN, Copenhagen, Denmark) RNA isolation as prescribed by CDC (*11*). We amplified the RESTV viral genome by targeting the NP gene developed by Sanchez et al*.* (*12*). The reaction was completed in a 20-µL reaction mixture containing 4 µL of RNA, 0.2 mmol/L of each dNTP, 1.6 mmol/LM MgSO_4_, 0.8 µL of SuperScript III RT/Platinum Taq enzyme mix (Invitrogen), and 0.5 µmol/L each of the primers RES-NP1 and RES-NP2. Thermocycling conditions were set to RT at 50°C for 30 min, inactivation and initial denaturation at 95°C for 3 min, then 45 cycles of 94°C for 30 s, 55°C for 30 s, 72°C for 30 s, then ending with a final extension step of 72°C for 5 min. Visualization of DNA bands was performed following electrophoresis in 2% agarose gels. Amplification of the latent gene from tissues was also undertaken by using the method adapted from Zhai et al. (*13*) using the Superscript Platinum Taq One-Step RT-PCR kit to generate 611-bp PCR products.

We then purified the PCR products and sequenced them with the BigDye Terminator v3.1 (ThermoFisher Scientific, Waltham, MA, USA) and analyzed in a 3500 Series Genetic Analyzer (Applied Biosystems, Foster City, CA). Assembled sequencing results were subjected to BLAST alignment (https://www.ncbi.nlm.nih.gov/BLAST), which confirmed the identity of the sequences as RESTV.

### Molecular Detection of Measles Virus

A TaqMan RT-PCR assay, distributed by CDC (*14*), was performed to detect MV RNA from macaques in the quarantine facility. Amplification of the MV RNA from sample DrpZ2-10B-G was undertaken using the HEN_RES_MOR primers described by Tong et al. (*15*) and produced a product of the expected size, which was sequenced.

### RESTV Isolation from Tissues

We attempted virus isolation in the liver; spleen; axillary lymph nodes (from DrpZ1-103A-K, DrpZ5-2B-F, and DrpL7-7D-A), and axillary, cervical, inguinal, and mesenteric lymph nodes (from DrpZ2-10B-G). Tissues were homogenized in PBS for 30 s with silicon carbide beads, centrifuged for 5 min, and the supernatant was added to a flask of semiconfluent Vero C1008 cells in PC4 containment at the CSIRO Australia Animal Health Laboratory, Victoria, Melbourne, Australia. For detection of virus replication, we performed 3 blind passages, monitored for CPE, and used a real-time PCR specific for NP of RESTV (J.S. Towner and P. Rollin, US Centers for Disease Control and Prevention, pers. comm., 2009 May 1).

### Next-Generation Sequencing of Isolates

We performed next-generation sequencing on the supernatant from passage 2 of the isolates from the axillary lymph nodes following TRIzol purification of RNA by using Direct-zol RNA Miniprep Kit (Zymo Research, Irvine, CA, USA). RNA was transcribed to cDNA by using random primers and SuperScript III reverse transcription (ThermoFisher SuperScript III First-strand Synthesis System), and then cDNA was prepared with Klenow (Promega, Madison, WI, USA). We used the Illumina Nextera XT library preparation kit to prepare dual barcoded libraries for 150-bp paired-end sequencing on the MiSeq system (Illumina, Scoresby, Victoria, Australia).

**Figure 1:**
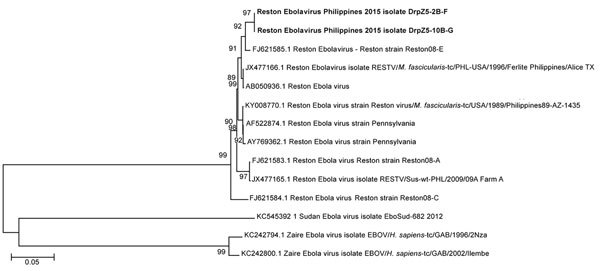
Phylogenetic tree (neighbor-joining) of the full genomes of Ebola viruses and comparison to the Reston 2015 viruses DrpZ52BF (GenBank accession no. MF540570) and DrpZ210BG (GenBank accession no. MF540571) produced by using MEGA 6 software (https://www.megasoftware.net). Bold text indicates the genomes being sequenced. Numbers along branches indicate bootstrap values. Scale bar indicates nucleotide substitutions per site.

We assembled the genome sequence of DrpZ5-2B-F (GenBank accession no. MF540570) and DrpZ2-10B-G (GenBank accession no. MF540571) by using RESTV genome GenBank accession no. FJ621585.1 as a reference. We generated phylogenetic trees (neighbor-joining method) from the full-length sequence (Figure 1) by using MEGA 6 software (https://www.megasoftware.net).

## Results

### Serologic Analysis

**Figure 2:**
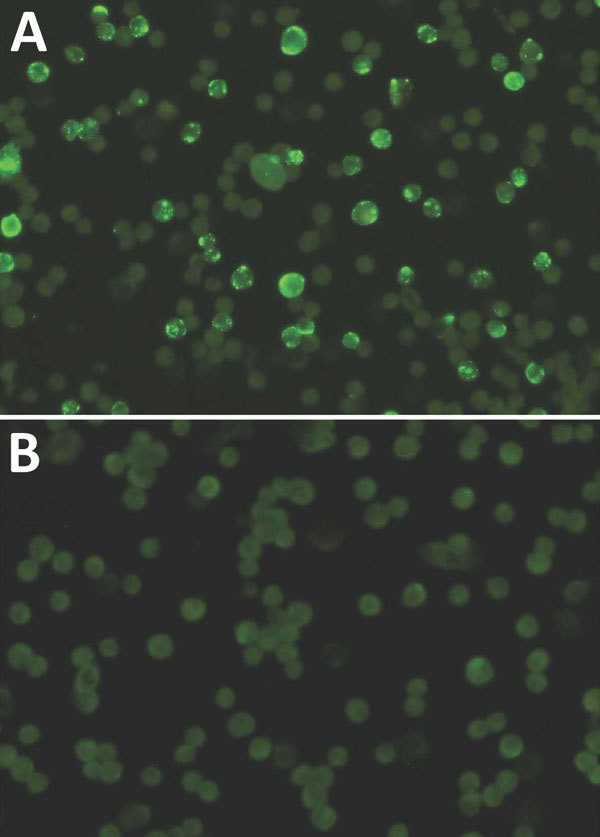
Immunofluorescence assay results of infected monkey serum A) characterized by granular staining pattern of HeLa cells and B) noninfected monkey serum. Original magnification ×400.

The RESTV ELISA showed that 10 of 174 samples (5.74%) from macaques were reactive for RESTV IgG antibodies (Table 1), all of which had an IFA titer >640 (Figure 2). These samples were also reactive in a Luminex assay, confirming the presence of IgG against RESTV. Of the 174 serum samples, 8 (4.59%) were reactive for MV IgM in the ELISA; 1 of the macaques (Identification no. DrpZ1-26D-B) was serologically positive for RESTV and MV antibodies (Table 1). Among those serologically positive for only MV IgM, 1 macaque (identification no. DrpZ2-10B-G) showed a positive result for a RESTV PCR in an autopsy sample (Table 2). The data indicated that among 174 macaques, 2 (1.1%) had a history of infection with RESTV and MV.

**Table 1:** *Reston ebolavirus* **a**ntibody-positive results in 174 cynomolgus macaque samples, the Philippines, August 2015*

Monkey ID	Date collected	Serologic analysis	
RESTV IgG	MV IgM
Drp6bL-27K-G	18	+	–
DrpL5–29D-B	18	+	–
DrpL7–7D-A†	27	+	–
DrpZ3–34C-E	27	+	–
DrpZ1–26D-B	27	+	+
DrpZ18–32B-E	27	+	–
DrpL3–3D-C	27	+	–
2DrpZ4–45C-F	27	+	–
13116B	27	+	–
DrpZ18–24B-B	27	+	–
DrpZ2–10B-G‡	27	–	+
DrpZ7–22A-F	27	–	+
DrpZ5–30D-A	27	–	+
DrpZ7–33A-I	27	–	+
DrpZ9–29A-I	27	–	+
2DrpZ5–36C-C	27	–	+
DrpZ8–12B-E	27	–	+

*ID, identification number; MV, measles virus; RESTV, Reston ebolavirus. †An autopsy sample of DrpL7–7D-A was positive for Reston ebolavirus PCR. ‡An autopsy sample of DrpZ2-10B-G was positive for both RESTV and MV PCR.

**Table 2:** Samples from cynomolgus macaques submitted for isolation of *Reston ebolavirus*, the Philippines, 2015*

Monkey ID	Sample type	Date collected	Isolation	RESTV PCR			MV PCR	
NP/L	GenBank accession nos., NP/L	L	GenBank accession no.
DrpZ1-103A-K	Liver	Aug 27	–	+/+	MG431944/MG431953	ND	ND
	Spleen	Aug 27	–	+/+	MG431945/MG431954	ND	ND
	Axillary lymph node	Sep 5	–	+/+	MG431952/MG431961	ND	ND
DrpZ5-2B-F	Cervical lymph node	Sep 5	–	+/+	MG431946/MG431955	ND	ND
	Axillary lymph node	Sep 5	+	+/+	MG431947/MG431956	ND	ND
	Inguinal lymph node	Sep 5	+	+/+	MG431948/MG431957	ND	ND
	Mesenteric lymph node	Sep 5	–	+/+	MG431949/MG431958	ND	ND
DrpZ2-10B-G†	Axillary lymph node	Sep 5	+	+/+	MG431950/MG431959	+	MF496232
DrpL7-7D-A‡	Axillary lymph node	Sep 5	–	+/+	MG431951/MG431960	ND	ND

*L, L gene; MV, measles virus; ND, not done; NP, nucleoprotein; RESTV, *Reston* *ebolavirus*; +, positive; –, negative. †Serum sample of DrpZ2-10B-G was RESTV IgG–/MV IgM+. ‡Serum sample of DrpL7-7D-A was RESTV IgG+/MV IgM­–.

Of the macaques from the 2 breeding facilities located in Oriental Mindoro and Rizal that supplied the animals to the quarantine facility, 10% were tested, as were personnel from both facilities. None of these personnel or macaques had detectable antibodies to RESTV.

### Virus Isolation and Molecular Analyses

RESTV was successfully isolated in Vero C1008 cells from the inguinal and axillary lymph nodes of DrpZ5-2B-F and the axillary lymph nodes of DrpZ2-10B-G (Table 2). Initial amplification of a 337-bp product of the partial NP gene from the liver, spleen, and lymph node tissue samples in 4 NHPs confirmed the presence of RESTV. Further amplification and sequencing of the partial L gene along with real-time detection further confirmed the RESTV infection. In all cases, Blast N revealed that the RESTV in this outbreak was most similar to the virus from the 2008 outbreak in swine (GenBank accession no. FJ624585.1) rather than the 1996 outbreak in NHPs.

The comparison of the whole genome sequencing of the 2 isolates DrpZ5–2B-F and DrpZ2-10B-G showed that there were 3 nucleotide differences. The first variation noted was in the NP gene (position 837 of the genome) of DrpZ5-2B-F, which resulted in a non-conservative amino acid change of a Thr (ACG) to a Lys (AAG) when compared with DrpZ2-10B-G and other RESTV isolates (Table 3). The change was also observed for all 4 specimen types of DrpZ5-2B-F (15–009–012), confirming that the change was not caused by passaging of the virus in cell culture. The second change noted was at position 4393 of the genome in the noncoding region between virus capsid proteins 35 and 40, and resulted in an adenosine for DrpZ5-2B-F and a guanine for DrpZ2-10B-G. The third variation was at position 10787 of the genome, and resulted in an amino acid change at position 162 of the VP24 protein from an Asn (AAC), which is common to all RESTV strains, to a Lys (AAA) for DrpZ5-2B-F. Therefore, DrpZ5-2B-F had 2 unique changes compared with other RESTV isolates. Both isolates showed 98% similarity to their closest RESTV strain (GenBank accession no. FJ621585.1).

**Table 3:** Nucleotide differences between the 2 *Reston* *ebolavirus* isolates, the Philippines, 2015*

Genome position	Reference sequence NC_004161.1	40 DrpZ5-2B-F	43 DrpZ2-10B-G	Amino acid position
837	C	A	C	NP (125)
	Thr	Lys	Thr	
4393	A	A	G	Noncoding (no amino acid change)
10787	C	A	C	VP24 (162)
	Asn	Lys	Asn	

*NP, nucleoprotein; VP, virus protein.

**Figure 3:**
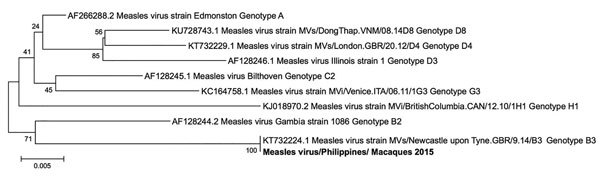
Phylogenetic tree (neighbor-joining) of the partial L gene (418 nt) of measles virus (GenBank accession no. MF496232) detected in macaques in 2015, produced by using MEGA 6 software (https://www.megasoftware.net). Numbers along branches indicate bootstrap values. Scale bar indicates nucleotide substitutions per site. Bold text indicates measles virus strain isolated in Philippines.

Because among 4 macaques that had a positive result by RESTV PCR, 1 (DrpZ2-10B-G) was serologically positive for MV (Table 1), we subjected a lymph node sample of this macaque to testing for MV by TaqMan RT-PCR to confirm the dual infection. As a result, we detected the MV genome, indicating that a dual infection occurred in this macaque (Table 2). Amplification of the partial L gene of MV RNA from sample DrpZ2–10B-G, followed by sequencing of the product and a BlastN of the sequence, revealed that the MV belonged to genotype B3, which had caused a large outbreak in the Philippines in 2014 (*16*) (Figure 3). MV RNA was also detected in 6 other macaques in the quarantine facility by using TaqMan RT-PCR.

## Discussion

In 2015, nineteen years after the last known epizootic occurrence of RESTV in macaques in the Philippines, we detected and confirmed the incidence of RESTV in macaques in a primate facility south of Manila by serologic and molecular testing. In spite of the long hiatus, RESTV was found in a controlled environment in which monkeys are systematically housed to avoid spread of diseases and to which no wild monkeys have been introduced. Personnel in the facility had no evidence of infection because no RESTV antibodies were detected.

We observed rats in cages in the primate facility that housed the primates being tested, indicating the potential for small animals to gain access to the facility. A recent study identified the microbat *Miniopterus schreibersii* as a possible reservoir of RESTV (*6*); therefore, this bat species and similar ones of this size may be the source of infection in the quarantine facility. If this is the case, improved biosecurity measures are warranted to limit the introduction of disease. However, we do not claim the bat species as the direct source of infection in 2015 outbreak. Because the facility building has its own anteroom with welded wire window screens, there is little likelihood that bats entered the facility.

Dual infections of RESTV and simian hemorrhagic fever virus (SHFV) in cynomologus monkeys have been reported in a facility in Reston, Virginia, and SHFV is the suspected causal agent for mortality in monkeys (*17*). Dual infections of RESTV and PRRSV in swine have been identified in the Philippines (*5*) and in Shanghai, China (*18*). In these cases, all of the RESTV-positive swine were coinfected with PRRSV. In contrast, we found in this study that 1 (ID: DrpZ1–26D-B) of the 10 macaques positive for RESTV antibody was also positive for MV antibody. Furthermore, another macaque (ID: DrpZ2–10B-G) was confirmed to have dual infection of RESTV and MV by using PCR. The results show similarities with dual infections such as SHFV and RESTV in macaques (*17*), or RESTV and PRRSV infections in swine (*5*). However, MV was not detected among most macaques positive for RESTV that died from the disease. Also, it remains unclear whether the MV infection supports an increase in RESTV replication in macaques. We found that 8 macaques had antibodies against MV, and 1 was MV PCR positive. Considering the risk for human-to-primate transmission (*19*,*20*), there is a possibility that MV infection in macaques is associated with human MV outbreak in the Philippines, although further studies are required to identify the mode of transmission of MV infection in macaques.

The RESTV sequences obtained were most similar to Reston-08-E from the Philippines 2008 outbreak in swine (*5*) (Figure 1). There were 3 nucleotide variations between the viral isolates that were sequenced, 2 of which in isolate DrpZ5–2B-F resulted in nonconservative changes in the NP and VP24 proteins that were unique when compared to all of the RESTV isolates sequenced. Because of the similarity with other Ebola viruses and the virus’ ability to infect humans, there is a concern that RESTV could mutate during passage through animals like macaques and cause an epidemic of disease in humans. Because it could mutate to pose health consequences for humans, continued surveillance is required to reduce the risk of transmitting Reston Ebola virus.
